# Effects of High-Phenolic Extra Virgin Olive Oil (EVOO) on the Lipid Profile of Patients with Hyperlipidemia: A Randomized Clinical Trial †

**DOI:** 10.3390/nu17152543

**Published:** 2025-08-02

**Authors:** Christos Kourek, Emmanouil Makaris, Prokopios Magiatis, Virginia Zouganeli, Vassiliki Benetou, Alexandros Briasoulis, Andrew Xanthopoulos, Ioannis Paraskevaidis, Eleni Melliou, Georgios Koudounis, Philippos Orfanos

**Affiliations:** 1 Department of Cardiology, 417 Army Share Fund Hospital of Athens (NIMTS), 11521 Athens, Greece; 2 Department of Hygiene, Epidemiology and Medical Statistics, School of Medicine, National and Kapodistrian University of Athens, 11527 Athens, Greece; vbenetou@med.uoa.gr (V.B.); phorfanos@med.uoa.gr (P.O.); 3 Cardiology Department & Department of Cardiac Catheterization, General Hospital of Messinia, 24100 Kalamata, Greece; manolismakaris@gmail.com (E.M.); koudounis@outlook.com.gr (G.K.); 4 Laboratory of Pharmacognosy and Chemistry of Natural Products, Department of Pharmacy, National and Kapodistrian University of Athens, University Campus, 15771 Zografou, Greece; magiatis@pharm.uoa.gr (P.M.); emelliou@pharm.uoa.gr (E.M.); 5 Department of Cardiology, Athens University Hospital Attikon, Medical School, National and Kapodistrian University of Athens, 12462 Athens, Greece; virginia_noa@yahoo.gr; 6 Department of Clinical Therapeutics, School of Medicine, National and Kapodistrian University of Athens, 11528 Athens, Greece; alexbriasoulis@gmail.com; 7 Department of Cardiology, University Hospital of Larissa, 41110 Larissa, Greece; andrewvxanth@gmail.com; 8 School of Medicine, National and Kapodistrian University of Athens, 11527 Athens, Greece; iparas@otenet.gr

**Keywords:** extra virgin olive oil (EVOO), hyperlipidemia, total cholesterol, low density lipoprotein (LDL), high density lipoprotein (HDL), lipoprotein a

## Abstract

Background/Objectives: Hyperlipidemia is a major risk factor for cardiovascular disease and atherosclerosis. Polyphenols found in polyphenol-rich extra virgin olive oil (EVOO) have been shown to possess strong antioxidant, anti-inflammatory, and cardioprotective properties. The present study aimed to assess the effects of two types of EVOO with different polyphenol content and dosages on the lipid profile of hyperlipidemic patients. Methods: In this single-blind, randomized clinical trial, 50 hyperlipidemic patients were randomized to receive either a higher-dose, lower-phenolic EVOO (414 mg/kg phenols, 20 g/day) or a lower-dose, higher-phenolic EVOO (1021 mg/kg phenols, 8 g/day), for a period of 4 weeks. These doses were selected to ensure equivalent daily polyphenol intake in both groups (~8.3 mg of total phenols/day), based on chemical analysis performed using NMR spectroscopy. The volumes used (8–20 g/day) reflect typical daily EVOO intake and were well tolerated by participants. A group of 20 healthy individuals, separated into two groups, also received the two types of EVOO, respectively, for the same duration. Primary endpoints included blood levels of total blood cholesterol, low-density lipoprotein (LDL), high-density lipoprotein (HDL), triglycerides, lipoprotein-a (Lpa), and apolipoproteins A1 and B. Measurements were performed at baseline and at the end of the 4-week intervention. Linear mixed models were performed for the data analysis. Results: The higher-phenolic, lower-dose EVOO group showed a more favorable change in total blood cholesterol (*p* = 0.045) compared to the lower-phenolic, higher-dose group. EVOO intake was associated with a significant increase in HDL (*p* < 0.001) and reduction in Lp(a) (*p* = 0.040) among hyperlipidemic patients in comparison to healthy individuals. Conclusions: EVOO consumption significantly improved the lipid profile of hyperlipidemic patients. Higher-phenolic EVOO at lower dosages appears to be more effective in improving the lipid profile than lower-phenolic EVOO in higher dosages.

## 1. Introduction

Extra virgin olive oil (EVOO), a major component of the Mediterranean diet, is one of the most extensively studied food categories due to its numerous beneficial effects on human health [1]. Its polyphenolic compounds, such as hydroxytyrosol, tyrosol, and their derivatives like oleocanthal, oleacein, oleuropein aglycon, and ligstroside aglycon, exhibit potent antioxidant, anti-inflammatory, and cardioprotective properties [2]. These properties contribute to improved vascular endothelial function and lipid profile, thus reducing risk factors for cardiovascular disease [3,4].

The lipid profile is a critical indicator of cardiovascular health, including parameters such as total blood cholesterol, low-density lipoprotein (LDL), high-density lipoprotein (HDL), triglycerides, lipoprotein a (Lpa), and apolipoproteins A1 (ApoA1) and B (ApoB). Among these, LDL, Lp(a), and ApoB have atherogenic action, whereas HDL and ApoA1 are considered anti-atherogenic. Pathological values of these markers are associated with the development of atherosclerosis, which is a major cause of cardiovascular disease [5,6]. Hyperlipidemia is one of the most prevalent modifiable risk factors for cardiovascular disease (CVD), the leading cause of morbidity and mortality worldwide [7,8]. According to the World Health Organization, elevated blood cholesterol is estimated to cause 2.6 million deaths and 29.7 million disability-adjusted life years (DALYs) annually [9]. In Europe, 50–60% of adults continue to have elevated total blood cholesterol (>193 mg/dL), with the burden high in middle-aged populations with poor dietary patterns or low physical activity, and approximately 75% of individuals at high cardiovascular risk do not reach LDL-C targets (<70 mg/dL), indicating a persistently high burden of unmanaged dyslipidemia and related cardiovascular risk [10,11]. Hyperlipidemia contributes significantly to atherosclerotic plaque development, ischemic heart disease, and stroke, making it a critical target for both pharmacologic and lifestyle-based interventions [12]. Therefore, the identification of dietary components, such as polyphenol-rich EVOO, that may favorably influence lipid metabolism is of growing scientific and clinical interest.

According to the European Union Regulation 432/2012, olive oils containing >5 mg of polyphenols (hydroxytyrosol and its derivatives, oleuropein, and tyrosol complex) per 20 gr belong to the category of olive oils with a “health claim” and protect blood lipids from oxidative stress [13]. Prior clinical trials, mainly in healthy volunteers, have demonstrated a dose-dependent effect of olive oil phenols on LDL oxidation and HDL levels. For example, the EUROLIVE trial showed that 25 mL of higher-phenolic EVOO (366 mg/kg) reduced LDL oxidation, while lower-phenolic refined olive oil (2.7 mg/kg) increased it [14]. However, these studies consistently administered the same oil volume, without testing whether a reduced quantity of higher-phenolic oil could match the effects of a higher-dose, lower-phenolic oil. Moreover, randomized controlled trials in hyperlipidemic subjects remain lacking, highlighting a critical gap that our study aims to address by comparing the phenolic concentration and dose in this population. Many consumers question whether it is more beneficial to consume higher doses of lower-phenolic-content olive oil or lower doses of higher-phenolic-content olive oil. Simultaneously, many producers are considering whether it is worthwhile to invest in producing olive oil with higher phenolic content. The present study provides, for the first time, an evidence-based answer to this question.

We hypothesized that the daily consumption of EVOO significantly improves the lipid profile in patients with hyperlipidemia. The aim of the present randomized clinical trial was to evaluate the effect of EVOO on specific lipid indices in hyperlipidemic patients, as well as to compare the impact of EVOOs that differ in phenolic concentration but match in total polyphenol intake.

## 2. Materials and Methods

### 2.1. Study Design and Participants

This study is a single-blind randomized clinical trial in which participants were blinded to the intervention group allocation. The two EVOO types were delivered in identical containers, and participants were unaware of the polyphenolic content or dosing rationale. Investigators, however, were aware of group allocation to ensure accurate dosing and compliance monitoring. It took place at the General Hospital of Messinia (Kalamata, Greece) between October 2021 and March 2022. The protocol fully complied with the Declaration of Helsinki (1975) and approved by the hospital’s Scientific Council (23/29 December 2020). All participants signed a consent form.

Participants were recruited as a consecutive series of outpatients diagnosed with hyperlipidemia, who attended the Outpatient Lipid Clinic of the Department of Cardiology at the General Hospital of Messinia during routine clinical visits. All individuals were screened for eligibility based on the inclusion and exclusion criteria described below and were invited to participate during routine follow-up visits. Fifty patients/volunteers were recruited and allocated into two intervention groups through stratified randomization based on age (groups separated according to cutoff value: 50 years) and LDL cholesterol levels (groups separated according to cutoff value: 150 mg/dL and/or cardiovascular risk). Randomization within each stratum (total of 4) ensured balanced group assignment where participants were allocated by sequential rotation. Additionally, 20 healthy individuals were recruited to compose an extra comparison group of individuals without hyperlipidemia, which were matched for age (mean age) and gender with 20 hyperlipidemic patients. In the final sample, the ratio of patients to healthy individuals was approximately 2.5:1, which occurred in practice without being set as a goal from the beginning but reflected the actual conditions of voluntary participation and availability.

### 2.2. Eligibility Criteria

The diagnosis of hyperlipidemia was based on medical history, clinical evaluation, and laboratory tests of each participant. Inclusion criteria were age ≥ 18 years and diagnosis of hyperlipidemia according to the criteria of the European Society of Cardiology [12].

Exclusion criteria included familial hypercholesterolemia or hypertriglyceridemia, chronic heart failure (New York Heart Association functional class stage ≥ 3), chronic renal failure with GFR < 15 mL/min/1.73 m^2^, and participation in other clinical intervention studies within the last 3 months.

### 2.3. EVOO and Intervention

The phenolic content of each EVOO was quantified using Nuclear Magnetic Resonance (NMR) spectroscopy [15], with one formulation containing 414 mg/kg and the other 1021 mg/kg of total phenolic compounds (including hydroxytyrosol and tyrosol derivatives). The intervention doses were selected to deliver equivalent amounts of polyphenols (~8.3 mg/day):

- Group 1: lower-phenolic content EVOO (414 mg/kg phenols) at a dose of 20 g daily.
- Group 2: higher-phenolic content EVOO (1021 mg/kg phenols) at a dose of 8 g daily.

**Figure 1:**
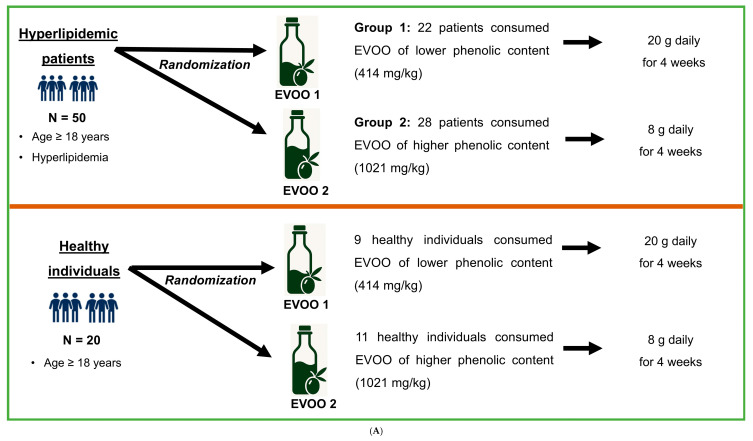

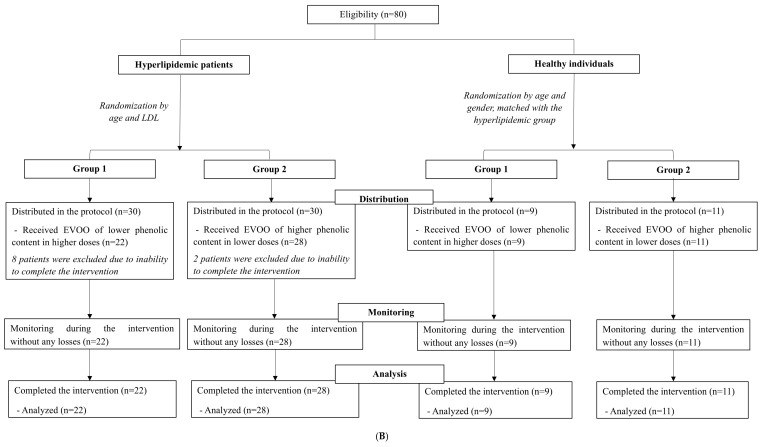
(**A**) Study design for (a) the comparison between hyperlipidemic patients and healthy individuals and (b) the comparison of EVOO with different phenolic content among patients with hyperlipidemia. (**B**) Flowchart depicting the participants’ distribution between hyperlipidemic patients and healthy individuals, as well as among subgroups. Group 1, hyperlipidemic patients/healthy individuals receiving lower-phenolic EVOO at a higher dose; Group 2, hyperlipidemic pa-tients/healthy individuals receiving higher-phenolic EVOO at a lower dose.

This design allowed us to compare the effectiveness of polyphenol concentration relative to fat volume while controlling for total polyphenol intake (Figure 1). These volumes were selected to be both nutritionally feasible and culturally acceptable, consistent with average olive oil consumption patterns in Mediterranean populations. The polyphenolic olive oil was consumed every morning at the same time in its original form, without any addition or mixing with other foods or liquids, and the participants were fasting for faster absorption of the phenols.

The two olive oils complied with chemical characteristics of the extra virgin category, and they both had the same ratio between total tyrosol derivatives and total hydroxytyrosol derivatives (Appendix B). The olive variety used in this study was Koroneiki for both olive oils to ensure similar lipid profile. The duration of the intervention was 4 weeks, during which participants were asked to maintain their usual diet and medication, without adding other products containing polyphenols.

Healthy individuals also consumed the two groups of EVOO for 4 weeks. To maintain consistency, the proportion of healthy individuals receiving each EVOO was similar to the respective proportion in patients (Figure 1A). Flowchart of this study is illustrated in Figure 1B.

Adherence was monitored through weekly telephone contact and the return of empty or partially used containers at the end of this study. Participants were reminded to consume the oil in its original form, every morning while fasting, and to avoid other polyphenol-rich foods or supplements.

Both EVOO types were provided in identical, dark glass bottles, labeled only with a study code. The oils were stored under controlled conditions (temperature: 15–18 °C, away from light and heat) to preserve freshness and phenolic content throughout the 4-week study period. The oils were used within three months of bottling, based on NMR-verified phenolic content.

As part of the single-blind design, participants were unaware of the phenolic content or volume rationale. Both groups received bottles of similar appearance and instructions. Investigators overseeing distribution and compliance were not blinded due to the need to assign dosage accurately.

This article is a revised and expanded version of a conference abstract entitled “Comparing the effects of different extra virgin olive oil (EVOO) doses and polyphenols concentration on the lipidemic profile and hemodynamic parameters in patients with hyperlipidemia”, which was presented at the ESC Preventive Cardiology 2024 conference, Athens, 25–27 April 2024 [16].

### 2.4. Endpoints

For the lipid profile assessment, fasting blood samples were collected at baseline and immediately after the intervention (4 weeks). Primary endpoints included total blood cholesterol, LDL-C, HDL-C, triglycerides, Lp(a), ApoA1, and Apo-B, assessed in a certified laboratory using standardized enzymatic methods.

Secondary analyses included differences between patient groups, as well as gender-specific differences.

### 2.5. Statistical Analysis

Power analysis was performed before the beginning of the clinical trial, using expected HDL changes from prior research [17]. We estimated that 60 patients with hyperlipidemia would be required to detect a significant increase of 7% (or approximately 4 mg/dL) in HDL cholesterol after the intervention, with a statistical power level of 0.8 and significance level of 0.05, after accounting for a dropout rate of 20% (~10 participants).

The normality of the distributions was tested with the Kolmogorov–Smirnov test for a sample size equal to or greater than 50 individuals, supported by visual inspection, in all cases, the normality was satisfactory. Descriptive data were expressed as frequencies and/or percentages for categorical variables and as mean ± standard deviation for continuous variables. The 2 independent-samples t-tests were used to compare mean differences, and the chi-Square test was used to compare proportions.

Although this study included only two time points per subject (baseline and post-intervention), linear mixed-effects models (LMMs) were chosen for their flexibility and ability to account for within-subject correlations. LMMs included fixed effects for time (before vs. after), group (patients vs. controls or EVOO group 1 vs. group 2), and their interaction (time × group) to assess whether the change over time differed significantly between groups. A random intercept for each participant was added to model inter-individual variability.

The general form of the model was

Y_ij_ = β_0_ + β_1_(Time_j_) + β_2_(Group_i_) + β_3_ (Time_j_ × Group_i_) + u_i_ + ϵ_ij_.

This modeling approach is appropriate even with two repeated measures per subject and is commonly used in clinical and nutritional studies where the primary aim is to test group-by-time interaction effects. It also accommodates unbalanced data (e.g., dropout) more effectively than repeated-measures ANOVA. The time × group interaction term was used to test whether the effect of the intervention differed between the comparison groups. Comparisons of the differences in parameters between the 2 independent groups and within each group were performed with post hoc analysis after Bonferroni correction, in order to address the problem of multiple testing and reduce type I error. To assess whether the addition of the interaction terms significantly improved model fit (full and partial models), the Akaike Information Criterion (AIC) was applied.

To explore sex-specific differences, LMMs were also fitted to further include the 3-way interaction (time × group × gender). While all statistical inferences were based on linear mixed models, repeated measures ANOVA was additionally used to produce visual summaries of time-dependent group differences.

All analyses were performed in R software (version 4.3.2), using R packages lme4 and emmeans for LMM.

## 3. Results

### 3.1. Participant Characteristics

The total sample of participants in this study consisted of 70 individuals: 50 patients with hyperlipidemia and 20 age- and sex-matched healthy individuals. Patients were further randomly allocated into two intervention groups, according to the phenolic content of the EVOO they received. Similarly, healthy individuals were randomly allocated into two groups, according to the phenolic content of EVOO they received. The final sample included 22 patients in Group 1 and 28 patients in Group 2. This imbalance resulted from the availability of participants in each stratum and from the dropouts in both groups due to inability to complete the program (Figure 1B). Participant adherence was 100% in both groups.

### 3.2. Comparison Between Patients with Hyperlipidemia and Healthy Individuals

Before the intervention, patients with hyperlipidemia and healthy individuals were demographically comparable (Table 1). The mean age was 52.2 ± 9.3 years for the patient group and 48.9 ± 8.7 years for the healthy group (*p* = 0.432) with similar gender distribution (24 men and 26 women in the patients and 8 men and 12 women in healthy individuals). The mean body mass index (BMI) was 27.2 ± 4.2 kg/m^2^ in the patients and 26.7 ± 5.1 kg/m^2^ in the controls (*p* = 0.865). As expected, the initial analysis of the lipid profile showed significantly higher values in the patient group compared to the controls. Specifically, total blood cholesterol was 224.8 ± 52.6 mg/dL in patients versus 170.7 ± 45.2 mg/dL in controls (*p* < 0.001). Similarly, LDL was increased in patients (142.7 ± 45.8 mg/dL) compared to controls (110.2 ± 32.7 mg/dL, *p* < 0.001). HDL and triglyceride values did not show statistically significant differences between the groups after the Bonferroni correction (*p* = 0.276 and *p* = 0.021, respectively).

**Figure 2:**
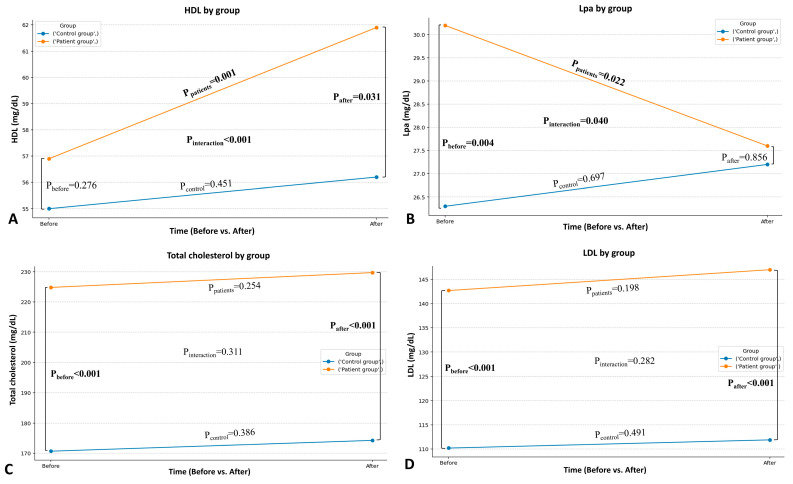

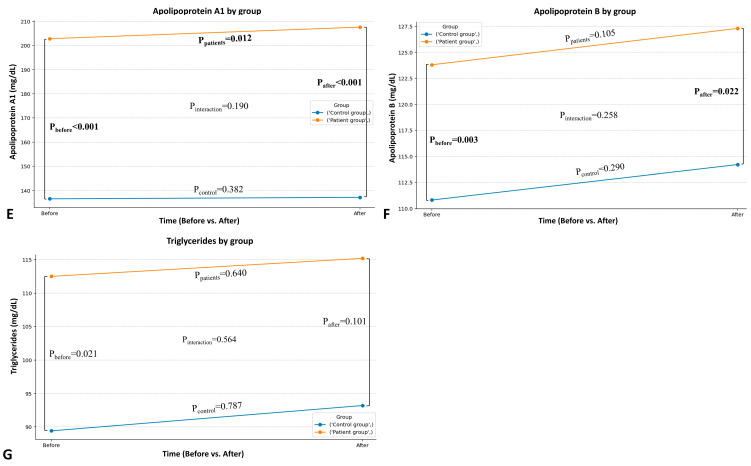
Comparison between the patient and the healthy group in HDL-C (**A**), Lp(a) (**B**), total blood cholesterol (**C**), LDL-C (**D**), apolipoprotein A1 (**E**), apolipoprotein B (**F**), and triglycerides (**G**) after the intervention.

Following the intervention, patients showed greater improvement compared to healthy individuals in specific blood lipid parameters. A statistically significant interaction between time and group (*p*_int_ < 0.001) was obtained for HDL (Figure 2A), indicating a greater increase in patients, while a moderate decrease in Lp(a) in patients, compared to the slight increase in healthy controls, was suggested (*p*_int_ = 0.040; Figure 2B). There were no statistically significant interactions as regards the remaining lipid parameters (Figure 2C–G).

### 3.3. Comparison Between the Two Groups of Hyperlipidemic Patients with EVOO of Different Phenolic Content

At baseline, the comparison of characteristics between the two groups of hyperlipidemic patients with different EVOOs did not reveal statistically significant differences in demographics or blood lipid parameters, making the groups suitable for subsequent comparisons (Table 2).

Table 3 presents the results obtained through a linear mixed model to evaluate the main effects of the time and intervention group and their interaction on each lipid parameter. The time × group interaction was marginally statistically significant in total blood cholesterol (β = −17.06; 95% CI: −33.29 to −0.83; *p* = 0.045), suggesting that the effect of the intervention was more beneficial in Group 2. No significant differences in HDL-C, LDL-C, Lp(a), ApoA1, or ApoB changes were observed between the two EVOO groups, although non-significant trends in favor of Group 2 (the high-phenolic formulation) were observed in most cases. The minimal differences in AIC values between full and reduced models further suggest that including the interaction term does not substantially improve model fit.

### 3.4. The Effect of Gender on the Intervention in Hyperlipidemic Patients

Although we explored the potential impact of gender on lipid responses to EVOO, no significant gender-related differences were revealed (Appendix A, Table A1).

## 4. Discussion

The present intervention study examined the effects of EVOO on the blood lipid profile of hyperlipidemic patients. The results showed that the consumption of EVOO had beneficial effects on the blood lipid profile of these patients, reducing atherogenic lipids such as lipoprotein α and increasing anti-atherogenic lipids such as HDL cholesterol, compared to the healthy population. Also, higher-phenolic EVOO in lower dosages had significant benefits in further reducing total blood cholesterol, compared to the lower-phenolic EVOO in higher dosages, highlighting further improvement of the lipid profile. According to the European Union Regulation 432/2012, olive oils containing more than 5 mg of polyphenols (hydroxytyrosol and its derivatives, oleuropein and tyrosol complex) per 20 g are entitled to bear a health claim regarding the protection of blood lipids from oxidative stress [13]. In our study, both EVOO formulations used exceeded this threshold substantially, with phenolic concentrations of 414 mg/kg and 1021 mg/kg, respectively, corresponding to more than 8 mg of phenols per 20 g in both cases. This provides a scientifically and legally justified basis for their selection. Importantly, this choice ensured that both oils met the criteria for health-promoting olive oil and allowed us to evaluate whether a higher concentration of phenols, when administered at a lower lipid dose, would yield superior lipid profile benefits compared to a lower phenolic oil at higher dosage, with matched total polyphenol intake.

Finally, we observed that gender did not play an important role in these lipid parameters, suggesting that there were no different responses to treatment among men and women. However, this finding should be interpreted with caution since this study was not powered for gender-specific analyses, and the relatively small sample size limited the ability to detect potential three-way interactions (time × group × gender) with sufficient statistical precision. As such, this analysis was exploratory in nature. The absence of statistically significant interactions does not necessarily imply a lack of effect but may reflect the limitations of the current sample size and study design. Larger studies specifically designed to assess sex-based differences are required to draw more definitive conclusions about the potential role of gender in EVOO interventions.

Several randomized controlled trials and meta-analyses support the conclusion that EVOO with higher polyphenol content confers greater therapeutic benefits than lower-phenolic or refined oils. These benefits include greater reductions in oxidized LDLs, improved HDL function, and enhanced antioxidant and anti-inflammatory activity. A recent systematic review and meta-analysis by Jabbarzadeh-Ganjeh et al. [18] showed that dose-dependent consumption of olive oil, regardless of type, appeared to be associated with an increase in HDL cholesterol. Specifically, an increase in olive oil consumption by 10 g/day was associated with a slight improvement in the lipid profile, including a decrease in LDLs by 0.04 mg/dL [(95% CI (−1.01–0.94); I^2^ = 80%] and an increase in HDLs by 0.22 mg/dL [95% CI (−0.01–0.45); I^2^ = 38%)]. However, a trend towards an increase of 0.79 mg/dL [95% CI (−0.08–1.66); I^2^ = 57%] was observed in total blood cholesterol, as was a trend towards an increase of 0.39 mg/dL [95% CI (−0.33–1.11); I^2^ = 7%] in triglycerides. The findings of our study are consistent with these results and, indeed, reinforce these findings, adding data on the reduction in Lp(a), which is an independent significant risk factor for cardiovascular disease [19,20]. However, there were also studies in the literature that did not show a significant improvement in the lipid profile of individuals after consuming EVOO. In particular, Castañer et al. in the EUROLIFE study did not find a significant increase in HDL in patients who consumed 25 mL of EVOO per day with a total phenolic content of 366 mg/kg for three weeks compared to those who consumed olive oil with a phenolic content of 2.7 mg/kg [14]. The difference in our results may be attributed to the fact that we used higher-phenolic EVOO compared to other studies that used olive oil with lower phenolic content. High polyphenol concentration may increase the efficacy in improving blood lipids, as supported by the studies of Covas et al. [21] and Visioli et al. [22], by protecting LDL particles from oxidation and reducing cardiovascular risk [23,24].

Hyperlipidemia, characterized by elevated levels and/or imbalance of blood lipids, such as LDL and HDL levels, is a major risk factor for cardiovascular disease [8,25]. It affects the homeostasis of the vascular endothelium, causing atherosclerosis and other vascular diseases [26,27]. High LDL concentrations lead to lipid oxidation and their accumulation in the endothelium, creating atherosclerotic plaques [28]. This process activates inflammatory pathways, forming atherosclerotic plaques. Vascular endothelial dysfunction, an early marker of atherosclerosis, is caused by an increase in reactive oxygen species (ROS), which leads to lipid oxidation and reduced NO production [29,30,31]. The result is monocyte activation, the expression of adhesion molecules such as intercellular adhesion molecule-1 (ICAM-1), and increased thrombogenesis [32].

Olive oil polyphenols, such as hydroxytyrosol, oleacein, and oleuropein aglycone, exhibit strong antioxidant and anti-inflammatory activity with protective properties on the vascular endothelium, improving the bioavailability of NO, which is critical for regulating vascular dilation. These compounds neutralize ROS and promote increased NO bioavailability, improving vascular endothelial function [33,34]. Hydroxytyrosol, oleuropein, and their derivatives scavenge ROS, reducing LDL oxidation and atherosclerotic plaque development [35,36]. Moreover, they suppress inflammatory adhesion molecules such as vascular cell adhesion protein-1 (VCAM-1) and ICAM-1, thereby reducing inflammation in vascular walls and reducing stiffness of large arteries and improving microcirculation in smaller vessels [37].

Lipids, such as LDLs and HDLs, have a direct physiological relationship with endothelial function. Elevated levels of LDLs and apolipoprotein B (ApoB), which are atherogenic, contribute to lipid accumulation in the vessel wall, causing endothelial dysfunction through inflammatory response and oxidative stress [38,39,40]. On the other hand, HDLs and ApoA1 have a protective effect, promoting cholesterol removal by macrophages and improving endothelial relaxation by increasing the bioavailability of NO [41]. Thus, the imbalance between atherogenic and anti-atherogenic lipids disrupts endothelial homeostasis, leading to increased cardiovascular risk.

Regarding the pharmacokinetics of phenolic compounds in humans, the mechanism of their absorption remains unclear. In the gastrointestinal tract, olive oil produces a micellar solution. Most of the phenolic compounds in EVOO pass through the oral cavity and stomach to reach the small intestine and colon without any modification [42]. Hydroxytyrosol and tyrosol have been shown to be the best absorbed phenolic substances in the intestinal tract in a dose-dependent manner, with an absorption rate of approximately 40 to 95% [43]. Equally important, various factors, such as food, can affect the absorption of phenolic compounds in EVOO. It has also been shown that these compounds are better absorbed when administered as a natural component of olive oil in liquid form, than in other forms or when co-administered with other foods such as water, yogurt, or even processed olive oil [44]. Interestingly, there appears to be a relationship between bioavailability and gender in animal studies. Specifically, male mice tend to have higher peak hydroxytyrosol concentrations than females, possibly as a result of differences in human enzymatic activity [45].

The enhanced efficacy of the higher-phenolic EVOO administered at a lower dose may be attributed to the higher polyphenol-to-lipid ratio, which is hypothesized to improve absorption efficiency. During digestion, olive oil forms micelles that incorporate both lipids and polyphenols [46]. When the same amount of polyphenols is delivered in a smaller lipid volume, the polyphenols may be more accessible at the micelle surface, facilitating more efficient uptake across the intestinal barrier. Additionally, lower lipid volumes may accelerate gastric emptying and release phenolic compounds more rapidly while minimizing interference from other fat-soluble components [47]. This may explain why equivalent doses of polyphenols, when delivered in a more concentrated lipid matrix, yield more pronounced lipid-lowering effects.

One of the strengths of this study was the randomized design and the allocation of patients into two intervention groups, based on the concentration of phenols in the olive oil. This grouping allowed for the investigation of the dose–response and the evaluation of the effectiveness of different doses. Furthermore, the inclusion of groups of healthy individuals to act as an extra comparison group increased the comparative power of this study, enhancing the ability to draw safe conclusions.

However, there were some limitations in our study. Firstly, the selection of participants was based on self-selection, which may lead to selection bias and further limit the generalizability of the results to the general population. Additionally, this study did not include a placebo control group, such as a refined olive oil with minimal or no polyphenol content. As such, we cannot entirely rule out that part of the observed lipid improvements may be attributable to the unsaturated fatty acid content of EVOO itself, rather than its phenolic constituents alone. Although our study was not designed to compare EVOO versus placebo but rather to assess the differential effects of two polyphenol-rich EVOOs with matched total phenol doses, future studies incorporating a true placebo group would be valuable to isolate the specific role of polyphenols in modifying lipid and inflammatory parameters. Another limitation of the current study was the relatively short 4-week duration of the intervention, which may not have captured the real benefits of the long-term effects of EVOO on the lipid status and vascular function of patients with hyperlipidemia, as well as healthy subjects. It is common for studies evaluating the effects of nutritional interventions to span three months or longer. The decision to assess outcomes at 4 weeks was made to ensure high compliance and capture early lipid responses, which have been previously reported in short-term interventions that used olive oil [17,21,22,48]. Due to the short duration of the intervention and lack of follow-up after cessation of EVOO intake, it remains uncertain whether the observed improvements in the lipid profile represent sustained changes or transient responses to polyphenol supplementation. Based on the known pharmacokinetics of polyphenols and prior evidence, it is plausible that continuous intake may be necessary to maintain these effects. Future studies with extended follow-up are warranted to evaluate the durability of these benefits after discontinuation. Moreover, the design of this study inherently couples phenol concentration with oil volume, and, as such, the effects of polyphenol bioavailability, fat content, and matrix effects cannot be completely disentangled. The superior lipid profile outcomes observed in the higher-phenolic/lower-volume group may result from improved intestinal absorption due to a higher polyphenol-to-lipid ratio, reduced overall lipid intake, or an interaction between concentration and delivery matrix. As a result, we suggest that future studies consider factorial designs to isolate the independent effects of polyphenol concentration and oil volume on lipid metabolism.

Our study was a single-blind study with the investigators not being blinded, a fact that could potentially introduce observation bias. However, we mitigated this risk by blinding participants and ensuring all outcome measurements were performed using standardized biochemical assays in an accredited laboratory, reducing the risk of subjective interpretation. Full double-blinding was not feasible due to logistical constraints and the need for accurate administration of different EVOO dosages. Moreover, due to the relatively limited sample size, especially when stratified by sex, this study may have been underpowered to detect potential sex-based differences in the lipid response, which should be explored in future, adequately powered studies. Additionally, although participants were instructed to maintain their usual diet, physical activity, and medication regimen, and avoid other types of EVOO or excessive use of plain olive oil during the intervention, no formal dietary or lifestyle assessment (e.g., 24-h dietary recall or food frequency questionnaire) was collected. This introduces a potential source of residual confounding. However, the randomized design should have helped ensure that such confounders were equally distributed between groups, reducing their potential impact on group comparisons, and all participants followed a Mediterranean-style diet. Future studies with longer follow-up periods could benefit from collecting dietary and lifestyle data to more rigorously assess adherence and adjust for potential confounders. Additionally, while adherence was monitored through regular follow-up and container return, self-reported compliance may still be subject to bias. Finally, our study did not assess inflammatory markers (e.g., CRP, IL-6, TNF-α), oxidized LDLs, or indicators of oxidative stress and endothelial function, which could provide mechanistic insights into the observed lipid profile improvements. Our group is currently conducting a complementary investigation that includes these biomarkers, and we anticipate that the forthcoming results will help elucidate the pathways by which high-phenolic EVOO exerts its beneficial cardiovascular effects.

It is important to note that all participants were recruited from a single center in Greece, and the EVOOs used in the intervention were derived exclusively from the Koroneiki olive variety, known for its high phenolic content. While this variety is widely cultivated and representative of high-quality Greek olive oils, polyphenol composition may differ across olive oil cultivars due to genetic and environmental factors. Therefore, extrapolation of our findings to other varieties, particularly those with lower or different phenolic profiles, should be made with caution. From a scalability perspective, our findings support the potential utility of producing EVOOs with high phenolic concentrations and favorable phenol-to-lipid ratios. Future studies across different populations and olive oil sources are warranted to validate and extend the applicability of our results.

## 5. Conclusions

The present study highlighted the positive effect of EVOO on the lipid profile of hyperlipidemic patients, including improvements in HDL and Lp(a) blood levels. Our findings indicate that higher-phenolic EVOO, even at a lower daily dose, provides superior benefits in improving the lipid profile of patients with hyperlipidemia compared to lower-phenolic EVOO administered at a higher dose with equivalent total polyphenol intake. This supports the hypothesis that the concentration and bioavailability of polyphenols are more important than the absolute lipid quantity consumed. These results also support the view that EVOO may constitute an important element of dietary interventions for the prevention and management of hyperlipidemia and related cardiovascular disease.

From a public health and commercial perspective, these results suggest that olive oil producers should consider investing in the development of high-polyphenol EVOO formulations, as they may offer greater therapeutic value at lower serving sizes. This approach could promote cardiovascular prevention strategies while helping consumers reduce overall caloric intake from fats without sacrificing health benefits. We would recommend that individuals consume EVOO with verified high polyphenol content, typically greater than 250–400 mg/kg. Labels or certifications indicating compliance with EU Regulation 432/2012 (i.e., ≥5 mg of hydroxytyrosol and derivatives per 20 g of oil) may serve as a useful guide. Selecting EVOOs produced from high-phenolic cultivars and stored in dark glass bottles away from heat and light can further ensure the potency and stability of phenolic compounds. Consumers aiming for cardiovascular benefits should focus not only on the quality and origin of the oil but also on daily, consistent intake, ideally in its raw, unheated form. However, broader long-term studies across different populations and olive oil varieties are warranted to confirm the scalability and generalizability of these findings.

## Figures and Tables

**Table 1:** Baseline demographic characteristics and lipid profile of 70 hyperlipidemic patients and healthy controls.

**Demographics**	**Patient Group**	**Healthy Individuals**	***p* Value** *
Number of participants (N)	50	20	
Gender (Males/Females)	24/26	9/11	0.954
Age (years)	52.2 ± 9.3	48.9 ± 8.7	0.432
BMI (kg/m^2^)	27.2 ± 4.2	26.7 ± 5.1	0.865
**Lipid Blood Profile**			
Total blood cholesterol (mg/dL)	224.8 ± 52.6	170.7 ± 45.2	**<0.001**
LDL-C (mg/dL)	142.7 ± 45.8	110.2 ± 32.7	**<0.001**
HDL-C (mg/dL)	56.9 ± 15.1	55.0 ± 12.3	0.276
Triglycerides (mg/dL)	112.5 ± 54.7	89.4 ± 25.2	0.021
Lp(a) (mg/dL)	30.2 ± 31.1	26.3 ± 19.2	0.460
ApoA1 (mg/dL)	202.8 ± 27.9	136.6 ± 24.5	**<0.001**
ApoB (mg/dL)	123.8 ± 32.5	110.8 ± 29.8	0.145

* Comparison of baseline values with *t*-test for independent samples and comparison of percentages with χ^2^ test. Abbreviations: BMI, body mass index; LDL, low-density lipoprotein; HDL, high-density lipoprotein; Lp(a), lipoprotein a; ApoA1, apolipoprotein A1; and ApoB, apolipoprotein B. Values in bold indicate statistically significant findings, taking into account the Bonferroni correction for quantitative indicators (*p* ≤ 0.007 = 0.05/7).

**Table 2:** Baseline demographic characteristics and blood lipid profile of the two intervention groups.

**Demographics**	**Group 1**	**Group 2**	*p* Value *
Number of patients (N)	22	28	0.433
Gender (Males/Females)	11/11	13/15	0.802
Age (years)	52.7 ± 8.0	51.8 ± 10.3	0.736
BMI (kg/m^2^)	26.5 ± 3.9	27.8 ± 4.5	0.289
**Lipid profile**			
Total blood cholesterol (mg/dL)	235.8 ± 59.3	216.1 ± 46.0	0.192
LDL-C (mg/dL)	151.9 ± 54.1	135.4 ± 37.4	0.232
HDL-C (mg/dL)	59.4 ± 14.4	55.0 ± 15.6	0.319
Triglycerides (mg/dL)	101.8 ± 44.2	120.9 ± 61.2	0.224
Lp(a) (mg/dL)	32.3 ± 30.8	28.5 ± 31.8	0.672
ApoA1 (mg/dL)	207.2 ± 28.6	199.3 ± 29.2	0.324
ApoB (mg/dL)	129.1 ± 37.6	119.6 ± 27.8	0.327

Group 1, patients receiving lower-phenolic EVOO at a higher dose; Group 2, patients receiving higher-phenolic EVOO at a lower dose; * comparison of baseline mean values with *t*-test for independent samples and comparison of percentages with χ^2^ test. Abbreviations: BMI, body mass index; LDL, low-density lipoprotein; HDL, high-density lipoprotein; Lp(a), lipoprotein a; ApoA1, apolipoprotein A1; and ApoB, apolipoprotein B. Bonferroni correction for quantitative indicators is performed (*p* ≤ 0.007 = 0.05/7).

**Table 3:** Beta coefficients (β) derived from linear mixed models to evaluate the interaction of time × group on blood lipid parameters.

Values	Estimator (β)	95% CI	*p* Value	AIC
**Total blood cholesterol (mg/dL)**				
(Intercept)	235.82	213.99–257.64	**<0.001**	Full model: 729.1 vs. Partial model: 727.4
Time (After)	14.46	2.31–26.60	**0.024**	
Group (Group 2)	−19.71	−48.87–9.45	0.191	
Time × Group (After × Group 2)	−17.06	−33.29–−0.83	**0.045**	
**LDL-C (mg/dL)**				
(Intercept)	151.86	132.37–171.36	**<0.001**	Full model: 989.2 vs. Partial model: 987.4
Time (After)	5.96	−3.95–15.86	0.245	
Group (Group 2)	−16.44	−42.49–9.62	0.222	
Time × Group (After × Group 2)	−2.88	−16.12–10.35	0.671	
**HDL-C (mg/dL)**				
(Intercept)	59.36	52.80–65.93	**<0.001**	Full model: 789.7 vs. Partial model: 788.0
Time (After)	5.73	1.66–9.80	**0.008**	
Group (Group 2)	−4.33	−13.10–4.44	0.338	
Time × Group (After × Group 2)	−1.37	−6.81–4.07	0.624	
**Triglycerides (mg/dL)**				
(Intercept)	101.77	77.78–125.77	**<0.001**	Full model: 1062.2 vs. Partial model: 1061.2
Time (After)	9.14	−8.18–26.46	0.306	
Group (Group 2)	19.12	−12.95–51.19	0.247	
Time × Group (After × Group 2)	−11.39	−34.53–11.76	0.340	
**Lp(a) (mg/dL)**				
(Intercept)	32.29	19.39–45.19	**<0.001**	Full model: 836.9 vs. Partial model: 835.0
Time (After)	−2.89	−6.07–0.29	0.081	
Group (Group 2)	−3.81	−21.04–13.43	0.667	
Time × Group (After × Group 2)	0.66	−3.59–4.91	0.763	
**ApoA1 (mg/dL)**				
(Intercept)	207.19	194.76–219.61	**<0.001**	Full model: 887.4 vs. Partial model: 885.4
Time (After)	5.13	−0.44–10.71	0.077	
Group (Group 2)	−7.91	−24.51–8.69	0.355	
Time × Group (After × Group 2)	−0.45	−7.89–7.00	0.907	
**ApoB (mg/dL)**				
(Intercept)	129.11	115.85–142.37	**<0.001**	Full model: 904.9 vs. Partial model: 904.1
Time (After)	6.02	−0.22–12.26	0.065	
Group (Group 2)	−9.51	−27.23–8.21	0.298	
Time × Group (After × Group 2)	−4.52	−12.86–3.82	0.293	

The category “Before” in time and the category “Group 1: patients who received EVOO of lower phenolic content at a higher dose” in the group are the reference values. Abbreviations: BMI, body mass index; LDL, low-density lipoprotein; HDL, high-density lipoprotein; Lp(a), lipoprotein a; ApoA1, apolipoprotein A1; and ApoB, apolipoprotein B. Values in bold indicate statistically significant findings.

**Figure A1:**
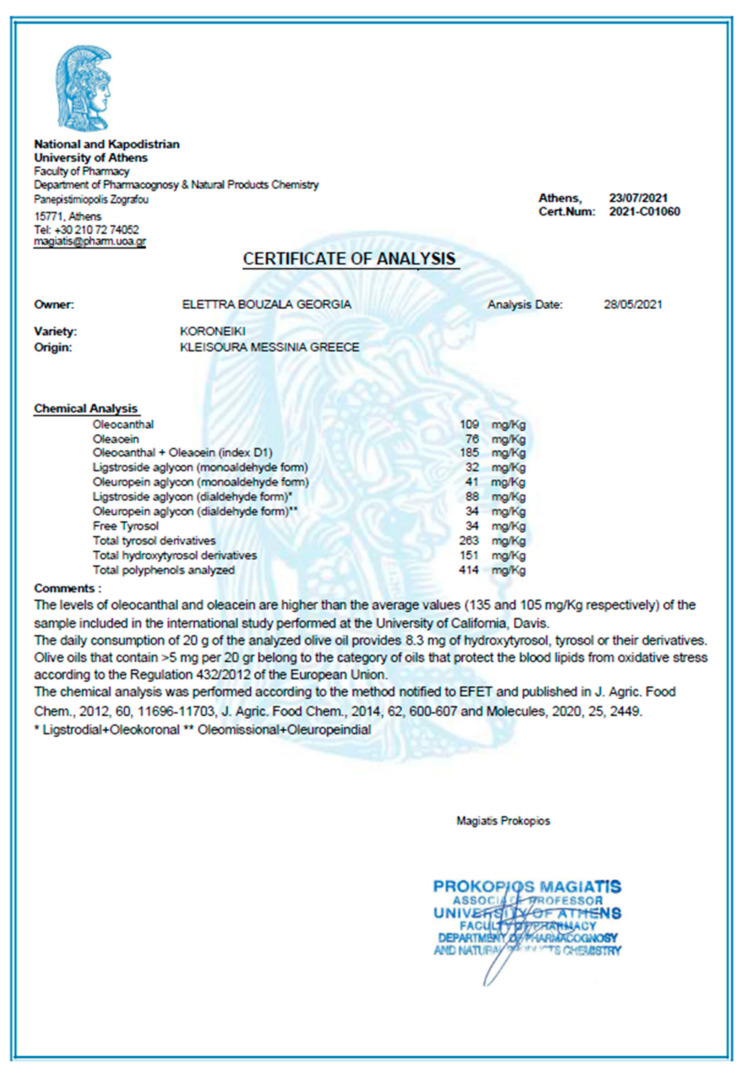
Certificate of analysis of EVOO of lower phenolic content.

**Figure A2:**
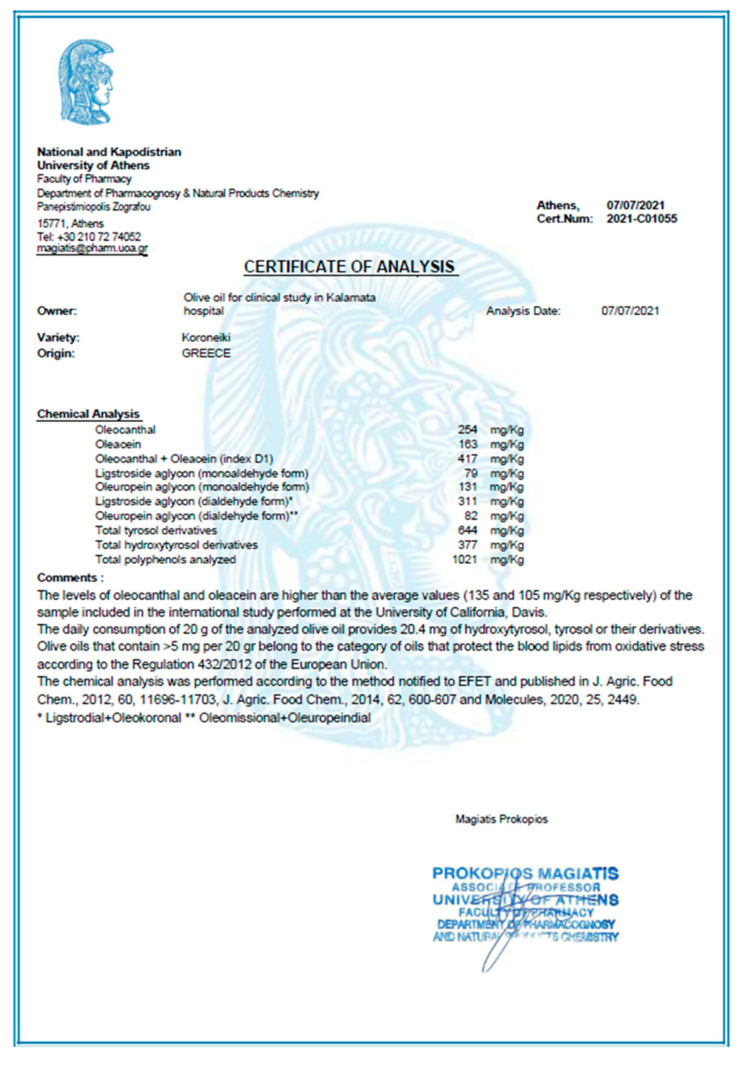
Certificate of analysis of EVOO of higher phenolic content.

## Data Availability

Data available upon request.

## Abbreviations

The following abbreviations are used in this manuscript:

EVOO	Extra virgin olive oil
LDL	Low-density lipoprotein
HDL	High-density lipoprotein
Lp(a)	Lipoprotein-a
ROS	Reactive oxygen species
ICAM-1	Intercellular adhesion molecule-1

## Appendix A

**Table A1:** Differences in the lipid profile parameters based on the impact of gender.

Lipid Profile Parameters	Time × Group × Gender Interaction	Group × Gender Interaction	Time × Gender Interaction
	*p* Value	*p* Value	*p* Value
Total blood cholesterol (mg/dL)	0.084	0.520	0.572
LDL-C (mg/dL)	0.138	0.408	0.474
HDL-C (mg/dL)	0.827	0.444	0.421
Triglycerides (mg/dL)	0.469	0.653	0.615
Lp(a) (mg/dL)	0.873	0.314	0.886
ApoA1 (mg/dL)	0.977	0.813	0.405
ApoB (mg/dL)	0.418	0.430	0.418

Abbreviations: LDL-C, low-density lipoprotein cholesterol; HDL-C, high-density lipoprotein cholesterol; Lp(a), lipoprotein a; ApoA1, apolipoprotein A1; and ApoB, apolipoprotein Β. The analysis is performed using linear mixed models to determine the existence of statistical significance in the interaction of time, gender, and group on each parameter of interest.
